# Sleep and Activity Patterns as Transdiagnostic Behavioral Biomarkers in Psychiatry: Longitudinal Observational Study From the DeeP-DD Study

**DOI:** 10.2196/81107

**Published:** 2025-11-14

**Authors:** Dylan Hamitouche, Tihare Zamorano, Youcef Barkat, Deven Parekh, Lena Palaniyappan, Sara Jalali, David Benrimoh

**Affiliations:** 1 Douglas Mental Health University Institute, 6875 LaSalle boulevard, Verdun, QC, H4H 1R3, Canada, 1 514-239-0428; 2 Department of Medicine, Faculty of Medicine and Health Sciences, McGill University, Montreal, QC, Canada; 3 Integrated Program in Neuroscience (IPN), Faculty of Medicine and Health Sciences, McGill University, Montreal, QC, Canada; 4 Department of Biochemistry, Faculty of Medicine, Université de Montréal, Montreal, QC, Canada; 5 Department of Psychiatry, McGill University, Montreal, QC, Canada; 6 Robarts Research Institute, Western University, London, ON, Canada; 7 Department of Psychology, McGill University, Montreal, QC, Canada

**Keywords:** actigraphy, digital biomarkers, digital phenotyping, physical activity, sleep

## Abstract

**Background:**

Despite widespread use of symptom rating scales in psychiatry, these tools are limited by reliance on self-report, infrequent administration, and lack of predictive power. This constrains clinicians’ ability to monitor illness trajectories or anticipate adverse outcomes like relapse. Actigraphy, a passive wearable-based method for measuring sleep and physical activity, offers objective, high-resolution behavioral data that may better reflect symptom fluctuations. Prior research has shown associations between actigraphy features and mood or psychosis symptoms, but most studies have focused on narrow diagnostic groups or fixed time windows, limiting clinical translation.

**Objective:**

This study aims to examine whether actigraphy-derived sleep and activity features correlate with psychiatric symptom severity in a transdiagnostic psychiatric sample, and to identify which features are most clinically relevant across multiple temporal resolutions.

**Methods:**

We present a feasibility case series study analyzing preliminary data from 8 outpatients (ages 18‐52 years) enrolled in the Deep Phenotyping and Digitalization at Douglas (DeeP-DD) study, a prospective transdiagnostic study of digital phenotyping. Participants wore wrist-based actigraphy devices (GENEActiv) for up to 5 months. Symptom severity was measured using a variety of self- and clinician-rated scales. We performed intraindividual Spearman correlations and interindividual repeated measures correlations across daily, weekly, monthly, and full-duration averages.

**Results:**

Intraindividual analyses revealed that later rise times were significantly associated with higher weekly 9-item Patient Health Questionnaire (PHQ-9) scores in participant 7 (ρ=0.74, *P*<.001) and participant 4 (ρ=0.78, *P*=.02), as well as higher weekly 7-item General Anxiety Disorder (GAD-7) scores in participant 7 (ρ=0.59, *P*=.03). While similar trends were observed at daily and monthly timescales, the weekly resolution yielded the most robust significance. Interindividual analyses showed that weeks with later average rise time correlated with higher PHQ-9 (*r*=0.48, *P*<.001) and GAD-7 scores (*r*=0.38, *P*=.03), with the PHQ-9 association remaining significant after Bonferroni correction (Bonferroni-corrected *P*=.02). Increased light physical activity was linked to lower PHQ-9 scores weekly (*r*=−0.44, *P*=.001) and monthly (*r*=−0.53, *P*=.01). Over the whole duration of the study, increased levels of sedentary activity were associated with lower GAD-7 scores (ρ=0.74; *P*<.001).

**Conclusions:**

Our findings highlight actigraphy-derived sleep and activity features, particularly rise time and physical activity, as promising transdiagnostic markers of psychiatric symptom burden. Their consistent associations across temporal scales and diagnostic groups underscore their potential utility for scalable, real-world clinical monitoring. Future work should validate these findings in larger cohorts and explore advanced analytical methods to capture circadian rhythmicity and symptom dynamics more precisely.

## Introduction

Despite decades of research into the neurobiology of various mental illnesses, there remains a lack of clinically reliable tools for identifying, monitoring, and staging most psychiatric conditions [1-4]. In major depression, best practices involve standardized rating scales as part of measurement-based care, which can help patients achieve remission more quickly [5-8]. These scales are clinically useful for tracking symptom severity and guiding treatment decisions. However, there are no widely accepted protocols for monitoring patients post-remission, and in severe mental illnesses such as schizophrenia, patient self-report is less reliable, while rating scales add to clinicians’ workload [9-12]. Moreover, rating scales may fail to capture what matters most to patients [13]—for instance, the commonly used 9-item Patient Health Questionnaire (PHQ-9) [14,15] does not distinguish between hypersomnia and insomnia. While rating scales are helpful for assessing illness status or guiding treatment adjustments, they are seldom used to predict events such as rehospitalization. This may be due in part to their limited predictive utility, as well as their infrequent and inconsistent use in clinical settings. Therefore, this gap in our ability to anticipate and prevent adverse outcomes highlights the need for more reliable, actionable predictors in clinical care.

In response, and in parallel with the rise of smartphones and wearables, interest has grown in “digital biomarkers” [16,17]—sensor-derived data collected passively by patient devices. Among those, digital monitoring and predictive biomarkers often use both smartphones and wearables, capturing data such as app usage, communication, location, light, movement, sleep, activity, and heart rate [18,19]. These tools have been used to infer mood states or behavior by combining multiple data streams—for example, call logs and GPS for sociability and mobility [19]. Integrating passive (sensor) and active (questionnaire) digital tools with clinical, historical, and biological data is one approach to “deep phenotyping”—a detailed characterization of patients. Among these tools, actigraphy, a wearable-based method for measuring rest-activity cycles, has emerged as particularly relevant. It provides objective, continuous data on sleep and activity, which are critical in conditions like bipolar disorder [20,21] and are often poorly captured by self-report [22].

Actigraphy has shown clinical relevance as a monitoring and predictive tool by detecting changes linked to symptom trajectories across psychiatric disorders. In schizophrenia, sleep disturbances captured by actigraphy may worsen symptoms via mood and attention-related pathways [23-25]. Studies show daily fluctuations in sleep and activity track symptoms, with physical activity linked to improved same-day mood but sometimes worse next-day symptoms [26]. In mood disorders, machine learning models using actigraphy can predict next-day depressive or manic episodes [27]. Depressed patients show reduced daytime activity, with age-specific sleep difficulties suggesting circadian disruption as a core mechanism [28-30]. Finally, in bipolar disorder, sleep and activity disturbances persist even during euthymia and are tied to episode onset, severity, and long-term outcomes [31-33].

Despite their promise, existing studies of actigraphy-derived sleep and activity features have often examined narrow diagnostic groups or short, fixed observation windows, limiting generalizability [34,35]. This limitation is significant given the multitude of available actigraphy measures, making it essential to identify which features demonstrate reliability and clinical relevance across diagnostic categories to optimize data collection and enhance clinical interpretation. Furthermore, since actigraphy data can be examined over multiple temporal scales, from days to months, understanding which measures maintain their utility across these timescales is critical. For instance, measures that are only informative over extended periods restrict their applicability for early or real-time clinical inference, as a substantial accumulation of data would be necessary before meaningful conclusions can be drawn. Addressing these limitations will improve the clinical utility and efficiency of actigraphy-based monitoring in psychiatric research and clinical practice.

This gap in the literature motivates the exploration of actigraphy-derived sleep and physical activity features as candidate monitoring and predictive biomarkers that transcend diagnostic categories and temporal scales. As part of the Deep Phenotyping and Digitalization at Douglas (DeeP-DD) project, a prospective transdiagnostic study aiming to identify clinically useful, scalable, and interpretable digital markers of mental illness, we present a case series study aiming to demonstrate the feasibility of actigraphy-based monitoring across diagnostic categories and temporal scales. We report data completeness rates, highlight the relevance of actigraphy for tracking subjective mood within and between individuals, and explore early signals of clinical relevance across multiple timescales in a real-world, diagnostically heterogeneous sample. Identifying robust, objective markers of illness could support clinical psychiatry by reducing reliance on disorder-specific tools and providing utility even when diagnostic clarity is limited, such as in youth mental health populations.

## Methods

### Overview

DeeP-DD is an ongoing feasibility study at the Douglas Mental Health University Institute in Montreal, Canada. Its objective is to test the feasibility and acceptability of multimodal digital phenotyping in a realistic transdiagnostic clinical population, including individuals with early psychosis, schizophrenia or schizoaffective disorder, bipolar disorder, anxiety, unipolar depression, and other conditions such as personality or substance use disorders. The study evaluates a wide range of digital tools, including actigraphy, smartphone sensors, ecological momentary assessments, and self-report questionnaires, and aims to develop interpretable “return of results” clinical reports to support personalized care.

DeeP-DD is designed as a feasibility and demonstration study rather than to test specific quantitative hypotheses. The planned target sample size is approximately 50 participants per diagnostic group (psychosis, bipolar disorder, depression, and personality or addiction disorder), for a total of about 200 participants. This sample size was chosen to enable assessment of transdiagnostic feasibility and data completeness across multiple modalities. As data collection progresses, analyses across all DeeP-DD components will inform the required sample size for a future interventional trial evaluating the impact of a combined digital phenotyping intervention and personalized feedback report. In this study, we focus on preliminary actigraphy and questionnaire data from the initial pilot phase, as these modalities were available for most participants.

### Ethical Considerations

The study was approved by the research ethics board of the West-Central Montreal health authority (Project No. 2023‐816) and conducted in line with the Declaration of Helsinki and the Tri-Council Policy Statement. Participants met with researchers who explained the study before providing written informed consent. Informed consent was obtained from all participants, and those who agreed to provide actigraphy data were provided with a GENEActiv wristband (Activinsights, Cambridge, UK) and given instructions on its use. Participant compensation was explicitly tied to engagement with study procedures to encourage adherence: participants received CAD $5 (US $3.55) per week for consistently wearing the actigraphy watch and CAD $7 (US $4.96) per week for completing the questionnaires. This approach was designed to motivate sustained participation. While this was a small compensation per week, it is still compensation, which would not be available in clinical settings; while this is fairly standard in research designs, the reader should keep this in mind when interpreting our results, especially with respect to data contribution rates. Participants provided written informed consent prior to enrollment. All data were deidentified and stored on secure servers, accessible only to authorized study personnel. No clinical care decisions were influenced by participation.

### Participant Recruitment

Participants are outpatients recruited from the Clinical High-Risk for Psychosis Clinic, First-Episode Psychosis Clinic, and the Bipolar Disorder Clinic; 1 patient with a primary personality disorder diagnosis was recruited from the general Neuropsychiatry Clinic. The study was advertised by clinicians directly to their patients during routine clinical visits and through posters displayed in the clinics. Participants are referred to the study by their clinicians or self-referred to the study after seeing study advertisements at their clinic.

### Data Collection

Patient- and clinician-rated questionnaire data were collected using the REDCap platform [36]. For every participant, a medical chart review was performed at the Douglas Mental Institute assessing overall symptomatology, medication use, and other clinically relevant information. Actigraphy data were extracted from the GENEActiv wristbands using GENEActiv PC Software (version 3.3; ActivInsights) [37], a validated approach to monitor sleep in adults [31-33]. The REDCap questionnaires included in the analyses were the PHQ-9 [14], which assesses depressive symptoms; 7-item General Anxiety Disorder 7 (GAD-7) [38], which evaluates anxiety severity; Clinical Global Impression - Severity (CGI-S) [39], which measures overall illness severity; Scale for the Assessment of Positive Symptoms (SAPS) [40], which examines psychotic symptoms; and Scale for the Assessment of Negative Symptoms (SANS) [41], which assesses deficits in normal emotional and behavioral functioning. The SAPS, SANS, and CGI-S were clinician-rated, while the PHQ-9 and GAD-7 were self-reported. The PHQ-9 and GAD-7 were administered weekly, the SAPS and SANS monthly, and the CGI-S at each clinical visit. Actigraphy data were inaccessible to participants and clinicians during the monitoring month but were made available to both after each monthly monitoring period concluded. As this was a real-world clinical feasibility study, both the timing of clinician-administered questionnaires and return of results depended on clinical follow-up schedules and as such were subject to variation in timing.

### Actigraphy Data Processing

Sleep and physical activity features extraction were processed from the raw movement data using GENEActiv default R markdown analysis tools [42]. Participants wore the actigraphy device for 1 month at a time (the length of a single charge), after which it was replaced with a newly charged device. The sleep features extracted and included in the analyses were as follows: total sleep time, sleep efficiency (ie, time spent asleep divided by time spent in bed), number of active periods per night, median length of those active periods, sleep onset time, rise time, day-to-day sleep onset time variability, and day-to-day rise time variability. The GENEActiv PC Software provided the daily step count and activity modes classified as “sedentary,” “light physical activity,” “moderate physical activity,” and “vigorous physical activity” [37]. We excluded vigorous activity from our analyses, as participants spent very little time in this mode (often less than 5 min per day), and these brief episodes were likely due to artifacts or misclassification rather than true vigorous exertion. We also included the daily duration of actigraphy nonwear in our analyses, as these periods may reflect participant behavior or symptom fluctuations and could provide meaningful insights into health status [43].

No filtering was performed on these features to maintain a straightforward preprocessing pipeline and preserve all available data, reflecting the exploratory nature of our analyses and the intent to apply these data in routine clinical practice using existing analysis tools. Due to the limited sample size, missing data were not imputed to avoid introducing bias. Actigraphy features and questionnaire scores were averaged by time unit (daily, weekly, monthly, and overall duration of study) to enable consistent comparisons between behavioral data and psychiatric outcomes across multiple time scales. Days with no data output despite the participant wearing the watch were excluded, as these were considered to result from sensor malfunction. To simplify preprocessing, enhance translatability, and given high participant compliance, we considered a day valid if any actigraphy data were recorded, and a week or month valid if it contained at least 1 such day. The results from sensitivity analyses using stricter thresholds (≥3 or ≥4 valid days for a valid week, and ≥10 or ≥15 valid days for a valid month) are available in Multimedia Appendix 1.

### Statistical Analyses

We conducted analyses at the intraparticipant level to capture how actigraphy features relate to psychiatric symptoms within each individual over time. For each participant, we computed Spearman rank correlations between actigraphy features and questionnaire scores at daily, weekly, and monthly resolutions, but only if at least 5 data points were available to ensure sufficient data for a stable and interpretable correlation estimate. This nonparametric method was chosen due to its robustness to nonlinear associations and ordinal or skewed data distributions. Correlations were computed independently for each participant and time scale. Bonferroni correction was applied across all tests to control for multiple comparisons.

Then, we performed analyses at the interparticipant level to identify consistent associations across the sample, accounting for repeated measures within individuals [44]. To do so, we used repeated measures correlation (Pingouin package, Python) [45] to assess associations between daily, weekly, and monthly actigraphy features and questionnaire scores across individuals, while accounting for nonindependence of within-subject data. Additionally, we computed Spearman correlations across participants using data aggregated over the full study period to capture stable between-subject associations and overall group-level trends not dependent on repeated measurements. To ensure reliable estimation, only correlations with at least 10 data points for daily analyses and 4 for weekly and full-study summaries were included, as interindividual analyses provided more data than intraindividual ones. These thresholds were chosen to balance data availability and reliability.

## Results

### Description

Data were collected from 8 participants, yielding a combined total of 33 months of actigraphy recordings. Participants were aged 18-30 years, included both males and females, and represented 3 primary psychiatric diagnoses with various comorbidities (Table S1 in Multimedia Appendix 1). Most participants met criteria for a first episode of psychosis, except for participant 5, who had a primary personality disorder, and participant 14, who had a primary bipolar disorder. Primary diagnoses included schizophrenia spectrum disorders (eg, schizophrenia, schizoaffective disorder), bipolar disorder, and personality disorder. Comorbid conditions commonly included depression, anxiety, attention-deficit or hyperactivity disorder, posttraumatic stress disorder, and personality disorders. Participants differed in clinical severity and care utilization: participants 4 and 8 had histories of hospitalization and multiple emergency room visits, while others (eg, participants 1, 5, and 13) had no acute care history. Medication use ranged from none (patient 1) to complex regimens involving antipsychotics, mood stabilizers, antidepressants, and stimulants (eg, participants 5 and 8) (Table S1 in Multimedia Appendix 1). Participants 1 and 6 did not consent to complete self-reported questionnaires.

**Figure 1.**
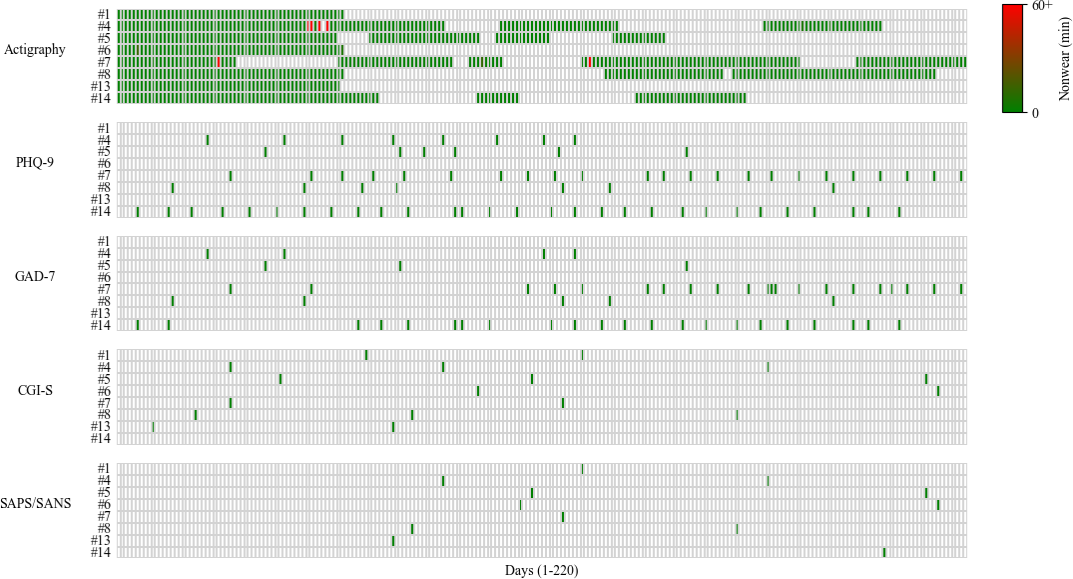
Visualization of actigraphy compliance and questionnaire completion across participants. Each green box represents a day with completed data. In the actigraphy panel, a color gradient indicates compliance (with green representing perfect wear and red indicating high nonwear of the GeneActiv device), while gray areas indicate days without data, which may result from sensor malfunction, participants returning the device, or the device being unavailable due to maintenance or the device being given to another participant due to a limited number of devices. Day 1 marks the start of wristband use for each participant, and the final day corresponds to the last day of actigraphy recording across all participants. Participant numbers on the y-axis are not sequential, as they represent individual patient IDs. CGI-S: Clinical Global Impression–Severity; GAD-7: 7-item General Anxiety Disorder; PHQ-9: 9-item Patient Health Questionnaire; SANS: Scale for the Assessment of Negative Symptoms; SAPS: Scale for the Assessment of Positive Symptoms.

The mean number of days of actigraphy data collected per participant was 110 (SD 43), ranging from 58 to 156 days. A total of 57 days were missing due to sensor malfunction, distributed across 3 participants (5, 7, and 14). Any additional missing days were due to the device being rotated among participants. Compliance with wearing the GeneActiv sensor was very high (97.27% of days showed no nonwear time; mean 1.58 min, median 0 min, SD 22.4 min). Across all recorded days of actigraphy (n=843), 820 showed no nonwear time, 23 had some nonwear time, and only 5 exceeded 1 hour of nonwear (maximum 9 h). On average, a valid week included 6.16 (median 7.00, SD 1.65, range 1‐7) days of data, while a valid month included 19.65 (median 22.00, SD 9.50, range 3‐31) days. Data missingness is reported in Figure 1 and Table 1.

**Table 1.** Summary of actigraphy adherence and questionnaire completion.

	Values, mean (SD)	Min	Max
Days of actigraphy	110.1 (40.4)	58	156
Missing days of actigraphy	7.1 (11.5)	0	35
Completed actigraphy (%)	95.4 (7.5)	78	100
PHQ-9^a^ completed	9.8 (11.0)	0	29
GAD-7^b^ completed	7.5 (9.5)	0	25
CGI-S^c^ completed	2.8 (1.2)	0	4
SAPS^d^ or SANS^e^ completed	2.1 (0.8)	1	3

a PHQ-9: 9-item Patient Health Questionnaire.

b GAD-7: 7-item General Anxiety Disorder.

c CGI-S: Clinical Global Impression–Severity.

d SAPS: Scale for the Assessment of Positive Symptoms.

e SANS: Scale for the Assessment of Negative Symptoms.

### Intraparticipant Patterns

**Figure 2.**
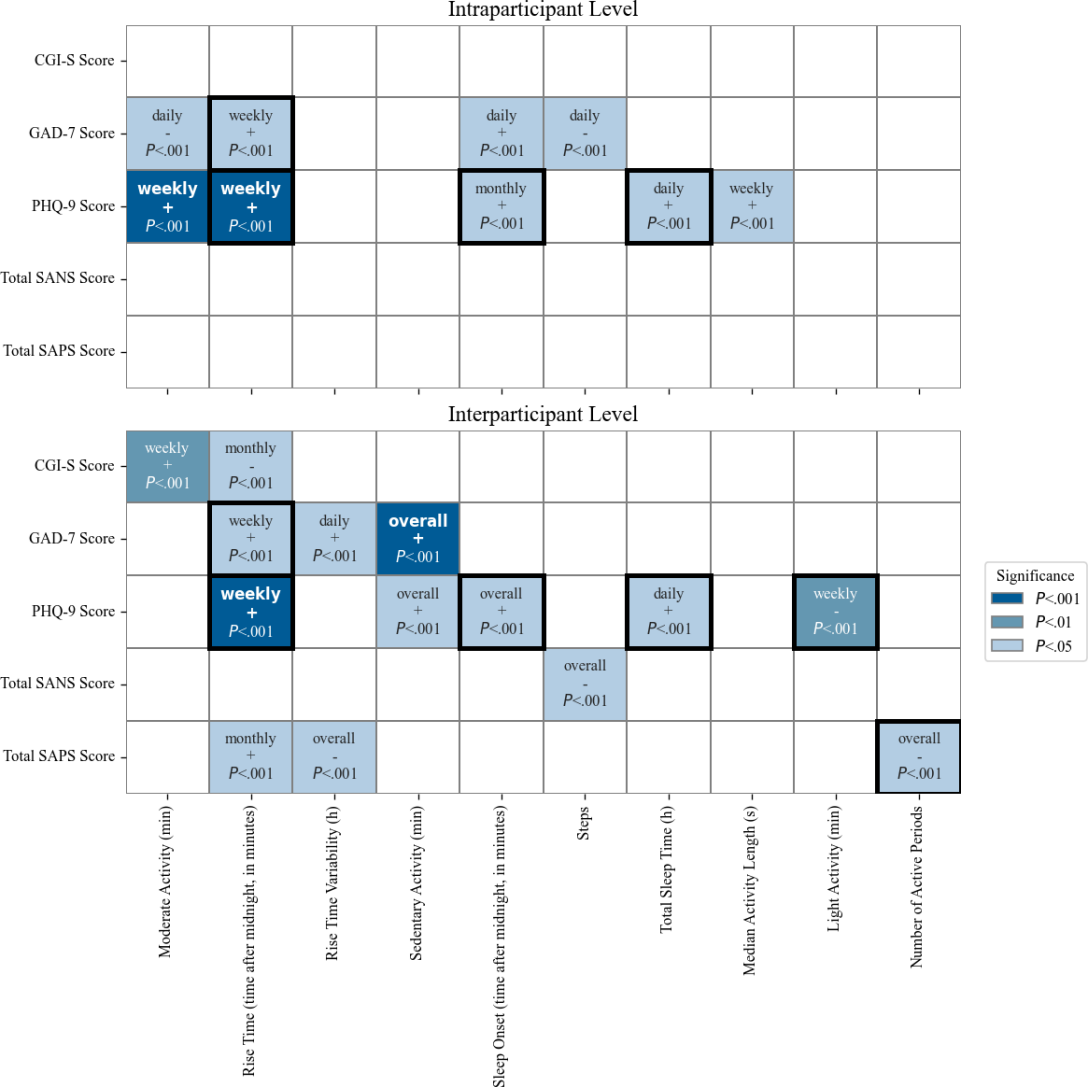
Heatmap of significant associations between actigraphy features and clinical questionnaires across multiple time scales at both intra- and interparticipant levels. “+” and “–” indicate the direction of the correlation. Cells with bold borders represent features with significant associations at multiple time scales (or across different participants for intraindividual analyses). Each cell displays only the most significant time scale. CGI-S: Clinical Global Impression–Severity; GAD-7: 7-item General Anxiety Disorder; PHQ-9: 9-item Patient Health Questionnaire; SANS: Scale for the Assessment of Negative Symptoms; SAPS: Scale for the Assessment of Positive Symptoms.

We analyzed same-day associations between questionnaire scores and actigraphy features within participants using Spearman correlations. In participant 7, higher GAD-7 scores were associated with a later sleep onset the night before (ρ=0.54, *P*=.04) and later same-day rise time (ρ=0.53, *P*=.04). However, these daily associations did not remain significant after Bonferroni correction. Figure 2 summarizes all significant associations established in this study.

On a weekly time scale, we found that for participant 4 (patient with a schizoaffective disorder and a personality disorder), a later rise time was associated with higher PHQ-9 scores, although the significance did not survive Bonferroni correction (ρ=0.8; uncorrected *P*=.02 Bonferroni-corrected *P*=.3). For participant 7 (patient with bipolar disorder, personality disorder, and a first-episode psychosis), a later rise time was associated with both higher PHQ-9 (ρ=0.74; *P*<.001) and GAD-7 scores (ρ=0.59; *P*=.03), although only the association with PHQ-9 stayed significant after correction (*P*=.007). We found that for participant 5 (patient who has a primary personality disorder), a longer time of moderate physical activity was associated with higher PHQ-9 scores (ρ=1.0; *P*<.001); see discussion below. This association correlates clinically, as it was noted in the participant’s clinical chart that they tended to go on long walks in the evening when feeling depressed. The higher level of significance for this participant is explained by the perfect correlation and limited number of overlapping data points for this participant (5 wk), which can inflate correlation coefficients.

Participant 7 showed that later average monthly rise time was associated with higher monthly average GAD-7 score (ρ=0.81; *P*=.05), while later average monthly sleep onset was associated with higher monthly average PHQ-9 scores (ρ=0.77; *P*=.04). None of these correlations remained significant after Bonferroni correction.

**Figure 3.**
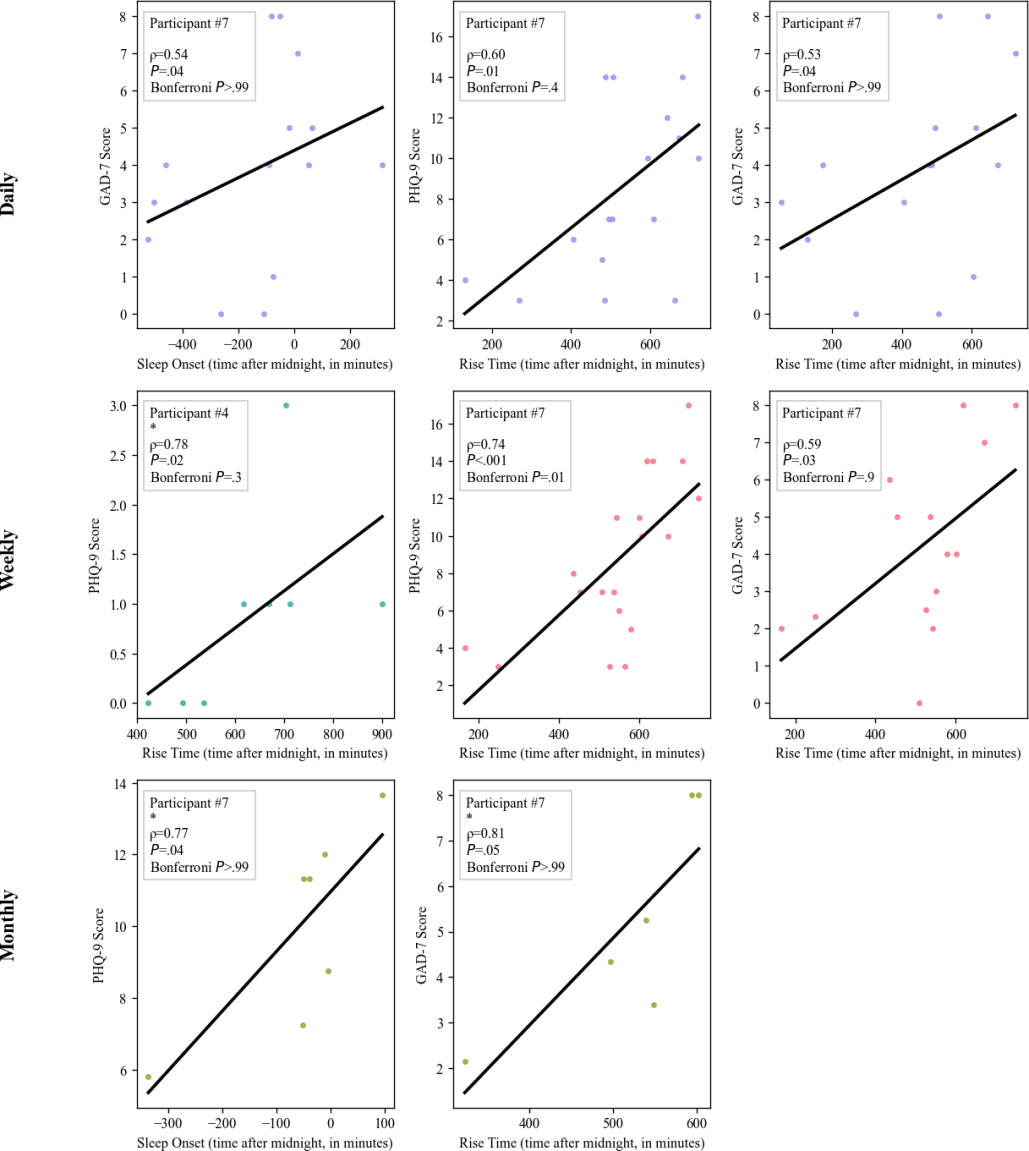
Significant intraindividual associations between actigraphy features and questionnaire scores across time scales. Asterisk (*) indicates fewer than 10 data points; Spearman correlation may be less reliable [46]. PHQ-9: 9-item Patient Health Questionnaire; GAD-7: 7-item General Anxiety Disorder.

In summary, several associations emerged between mood scores and sleep timing or physical activity. After correction, only weekly associations involving rise time and PHQ-9, and moderate activity and PHQ-9 (which was most likely idiosyncratic to participant 5), remained significant—highlighting rise time and activity patterns as potential behavioral markers of mood (Figure 3).

### Interparticipant Trends

Same-day associations at the interindividual level showed longer total sleep duration the night before (*r*=0.30; *P*=.03). Increased rise-time variability, defined as the absolute difference in rise time between consecutive days, was correlated with higher GAD-7 scores (*r*=0.39, *P*=.02). However, none of the same-day associations survived correction for multiple comparisons.

Interestingly, the intraparticipant associations between rise time, sleep onset time, and questionnaire scores remained consistent in our interparticipant repeated measures correlation analyses conducted at a weekly time scale, such that weeks with a later average rise time were associated with a higher GAD-7 (*r*=0.38; *P*=.03) and PHQ-9 score (*r*=0.49; *P*<.001). Furthermore, weeks of increased time spent doing light physical activity were associated with lower PHQ-9 (*r*=−0.44; *P*=.001). Only the association between PHQ-9 scores and rise time remained significant after Bonferroni correction (*P*=.02).

On a monthly time scale, increased light physical activity was associated with lower PHQ-9 scores (*r*=−0.53; *P*=.01). Later average monthly rise time was associated with both higher average CGI-S (*r*=−0.68; *P*=.05) and lower total SAPS (*r*=0.96; *P*=.04). The association with higher CGI-S appears contradictory to the rest of the study’s findings linking later rise time with better outcomes, but this is likely driven by participant clustering—specifically, participant 4, who had consistently later rise times but low CGI-S scores. However, none of the monthly associations remained significant after Bonferroni correction.

**Figure 4.**
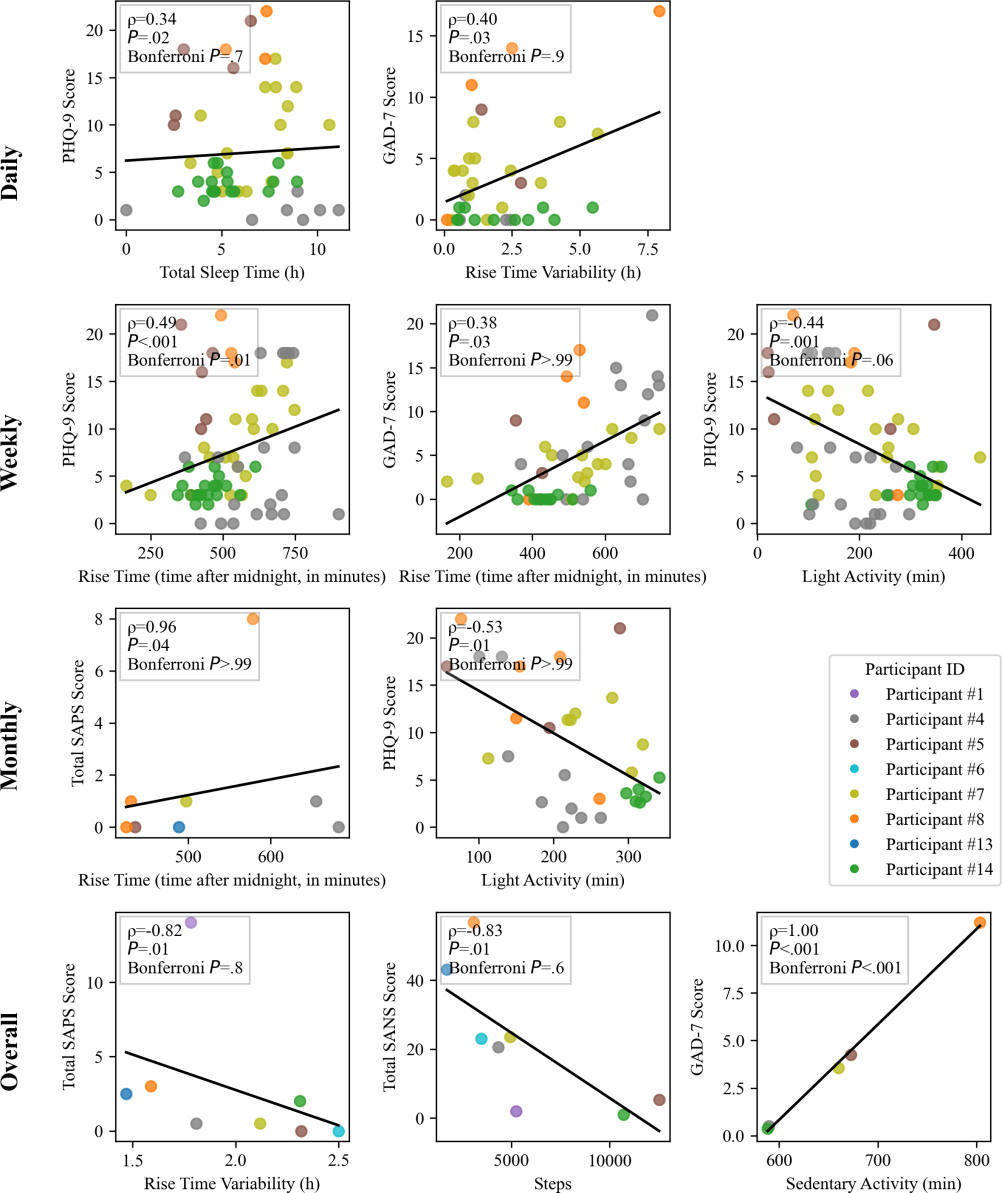
Significant interindividual associations between actigraphy features and questionnaire scores across time scales. Repeated measures correlations were conducted at the daily, weekly, and monthly time scales; Spearman correlations were used for overall averages across the full study duration. CGI-S: Clinical Global Impression–Severity; GAD-7: 7-item General Anxiety Disorder; PHQ-9: 9-item Patient Health Questionnaire; SANS: Scale for the Assessment of Negative Symptoms; SAPS: Scale for the Assessment of Positive Symptoms.

When comparing participants’ average questionnaire scores and actigraphy features collected over the study period (ranging from 1 to several months, depending on each participant’s enrollment date), Spearman correlation analyses showed that participants with a later average sleep onset had a higher PHQ-9 scores average (ρ=0.90; *P*=.04). Participants with more average daily time spent in sedentary activity had higher PHQ-9 score (ρ=0.90; *P*=.04) and GAD-7 score averages (ρ=1.00; *P*<.001); however, after Bonferroni correction, only the GAD-7 association remained significant (*P*<.001), though this unusually strong correlation should be interpreted with caution given the small sample size (Figure 4).

Sensitivity analyses using stricter validity thresholds (≥3 or ≥4 valid days per week, and ≥10 or ≥15 valid days per month) were conducted to assess the robustness of our findings and are available in Multimedia Appendix 1. These analyses showed similar patterns of associations, though with reduced statistical significance, as expected with decreased data availability and statistical power.

In summary, findings from interparticipant analyses aligned with intraparticipant results, showing consistent trends where symptom improvement was linked to earlier rise times, longer sleep duration, and increased time spent doing light physical activity, while symptom worsening was associated with increased sedentary behavior and delayed sleep onset. These associations were stronger at the weekly level, with some remaining significant after correction for multiple comparisons.

## Discussion

This case series, focused on the feasibility of the use of actigraphy in a realistic clinical setting, explored the relationship between sleep and activity patterns and psychiatric symptom severity across multiple diagnostic categories and time scales. Despite our small and heterogeneous sample, several meaningful intra- and interindividual associations emerged. Notably, sleep timing (particularly rise time) showed consistent associations with mood scores at the individual level, but also at the group level and at different time scales. While these results align with prior evidence linking delayed sleep-wake cycles to mood disorders and psychosis, our study is novel in showing these associations within a realistic transdiagnostic population and highlighting their persistence across different temporal scales [47,48]. This holds clinical significance, as it underscores the potential of sleep timing as a modifiable biomarker and intervention target for improving mood symptoms across a range of psychiatric disorders.

At the individual level, higher depressive and anxious symptoms were consistently associated with delayed circadian rhythm, observed at both daily and weekly time scales. For participant 5, who we know clinically to become more agitated when depressed, the presence of worse depressive symptoms during weeks of increased moderate physical activity likely reflects this pattern. This highlights the importance of accounting for individual clinical context when interpreting behavioral data. Interindividual analyses echoed these findings, showing that weeks with more severe depressive and anxious symptoms were also characterized by later rise times. The persistence of these associations after correction in this small, heterogeneous sample strengthens the case for delayed sleep-wake phase (ie, a regular sleep schedule that is considerably later than the conventional or desired time) as a transdiagnostic marker of psychiatric symptom burden. This characteristic has been previously linked with worse depressive symptoms in young adults [49] and worse outcomes in patients at clinical high risk of psychosis, as well as in those with early psychosis or schizophrenia [48,50], but we provide new evidence that these significant changes may occur across temporal scales and diagnostic groups [51,52]. We also found that during weeks and months of increased physical activity, participants reported less severe depressive and anxiety symptoms, consistent with findings from previous studies [53].

Same-day analyses, although not surviving correction for multiple comparisons, showed that shorter total sleep duration the night before, earlier rise time, and a rise time that has less variability from the previous day are each associated with lower same-day symptom severity. The unexpected association between total shorter sleep duration the preceding night and lower same-day symptom severity should be interpreted with caution, as the relationship was weak and largely driven by a small number of participants (notably participants 5 and 7). The sleep timing findings further support prior evidence that circadian phase is a key predictor of same-day mood episodes [26]. These trends point toward possible short-term responsiveness of mood and anxiety symptoms to daily behavioral patterns.

Additionally, when comparing participants’ data across the entire study period (ie, averaged across all months), those with later average sleep onset and more sedentary behavior tended to report worse symptoms, while those with higher average daily step counts reported fewer negative psychotic symptoms. Due to very high compliance with wearing the GeneActiv sensor (97.27% of recorded days of actigraphy showed no nonwear time), we did not find any significant associations between non-compliance and questionnaire scores.

Despite the promising findings described above, several limitations must be acknowledged. The small sample, as well as missing data due to sensor malfunctions and variable adherence to actigraphy and questionnaire protocols, may have limited statistical power, introduced bias, and reduced sensitivity to detect certain effects. Furthermore, it is important to note that inferring temporal causality from these observations remains challenging; incorporating ecological momentary assessments data could provide a more precise temporal resolution, thereby improving the ability to disentangle the directionality of the relationship between sleep timing and symptom trajectory [54]. In addition, the use of GENEActiv devices requires physical docking to download data, meaning that data are not available in real time. As such, real-time monitoring of adverse events or symptom burden (eg, hospitalizations) was not possible in this study. Alternative wearable devices with real-time data transmission capabilities may be better suited for future studies aiming to evaluate actigraphy as a biomarker for timely clinical intervention. However, the actigraphy data were able to be processed and included in return-of-results reports to clinicians and patients on a monthly basis, which was preliminarily found to be clinically useful; the process of developing these reports will be discussed elsewhere. Nonetheless, the consistency of our results across time scales and methods highlights sleep timing and physical activity as a promising behavioral biomarker in real-world psychiatric populations.

The preliminary findings from the DeeP-DD study reveal complex associations between sleep features and clinical symptoms across various diagnostic groups, time scales, and data collection methods within a realistic clinical population. Earlier sleep and wake times, along with higher physical activity levels, were consistently associated with better clinical outcomes, including lower anxiety and depressive symptoms, both within and between individuals and across multiple time scales. The weekly time scale was particularly interesting, as multiple associations involving both sleep timing and physical activity were significant across participants. Although some associations did not remain significant after correction for multiple comparisons, the consistent patterns observed at both the inter- and intraindividual levels align with prior literature and offer novel insights into which actigraphy metrics, for different durations, may serve as digital biomarkers of monitoring and prediction or psychiatric symptom trajectories in a transdiagnostic population. However, given the small sample size, these conclusions should be interpreted with caution. The next steps in our project involve further exploring these relationships using larger datasets to assess their consistency and investigate the longitudinal dynamics between sleep and symptom changes. Additionally, we aim to incorporate more sophisticated approaches such as Fourier transformations at an intraindividual level to capture rhythmicity and periodic patterns in sleep and activity data, which may reveal subtle disruptions in circadian cycles linked to symptom fluctuations [55]. These methods could help identify individual-specific signatures, ultimately informing personalized interventions.

## Supplementary material

10.2196/81107 Multimedia Appendix 1 Clinical and sociodemographic characteristics, and figures showing significant associations between actigraphy features and clinical questionnaire scores across multiple time scales, at both the intra- and interindividual level.

## Abbreviations

CGI-S: Clinical Global Impression–Severity
DeeP-DD: Deep Phenotyping and Digitalization at Douglas
GAD-7: 7-item General Anxiety Disorder
PHQ-9: 9-item Patient Health Questionnaire
SANS: Scale for the Assessment of Negative Symptoms
SAPS: Scale for the Assessment of Positive Symptoms
