# Supplementary material for: Sleep and Activity Patterns as Transdiagnostic Behavioral Biomarkers in Psychiatry: Longitudinal Observational Study From the DeeP-DD Study
Source: JMIR Form Res. 2025 Nov 14;9:e81107. doi: 10.2196/81107 (PMC12617961; doi:10.2196/81107)
Supplement: Multimedia Appendix 1 [file formative-v9-e81107-s001.pdf]

Table S1. Clinical and sociodemographic characteristics of participants. CHR: Clinical High Risk psychosis. Age was rounded down to the nearest decade to preserve confidentiality.[a-u]

Participant	Age	Sex	Employed	Clinic	Primary diagnosis	Diagnostic impression	Psychiatric Family History	Hospitalizations	ER Visits
#1	20's	F	No	CHR	CHR-P	CHR-P, Dissociative identity disorder, Complex trauma, Anxiety, potentially transferring to psychosis	MDD, Anxiety, BPD, SUD	0	0
#4	20's	M	No	FEP	SSD	FEP in the form of mania, most likely Schizoaffective disorder given negative symptoms, r/o concomitant mood disorder	Schizophrenia, Anxiety	1	1
#5	30's	M	Yes	Neuropsychiatry clinic	Mixed PD	Mixed personality disorder (clusters B and C), Gambling addiction	None	0	0
#6	20's	F	Yes	FEP	SSD	Schizophrenia,, Depression, Social anxiety	None	0	1
#7	20's	M	Yes	FEP	BD-1 with psychotic features	BD-1, ADHD, Mixed anxiety dis (GAD/SAD), SUD	None	0	1

						(cannabis), C-PTSD with dissociation, Panic attacks, Functional neurological disorder, r/o: personality dis (mixed with paranoia, narcissistic traits, OCDP), r/o: tourettes, r/o: OCD			
#8	10’s	M	No	FEP	SSD + ASD	Autism, Schizophrenia, MDD, likely gender dysphoria, ASD, Anxiety NOS, r/o ADHD	MDD, SAD, Schizophrenia, Bipolar Disorder	1	3
#13	20’s	F	No	FEP	SSD	Psychosis NOS, likely schizoaffective -depressive type, Mixed anxiety disorder (GAD, panic attacks), Tourette's syndrome (not currently, major focus of treatment), OCD traits, ADHD, Auditory processing	Schizophrenia	0	0
#14	50’s	F	Yes	Bipolar clinic	BD-2	BD-II, Anxiety disorder	Bipolar disorder, Depression, Anxiety disorder, Gambling addiction	0	2

ADHD: Attention-Deficit/Hyperactivity Disorder

ASD: Autism Spectrum Disorder

BD-I: Bipolar Disorder type I

BD-II: Bipolar Disorder type II

BPD: Borderline Personality Disorder

C-PTSD: Complex Post-Traumatic Stress Disorder

CHR: Clinical High Risk

CHR-P: Clinical High Risk for Psychosis with Positive symptoms

FEP: First-Episode Psychosis

GAD: Generalized Anxiety Disorder

MDD: Major Depressive Disorder

NOS: Not Otherwise Specified

OCD: Obsessive-Compulsive Disorder

OCDP: Obsessive-Compulsive Personality Disorder

PD: Personality Disorder

PTSD: Post-Traumatic Stress Disorder

r/o: Rule out

SAD: Social Anxiety Disorder

SSD: Schizophrenia Spectrum Disorder

SUD: Substance Use Disorder

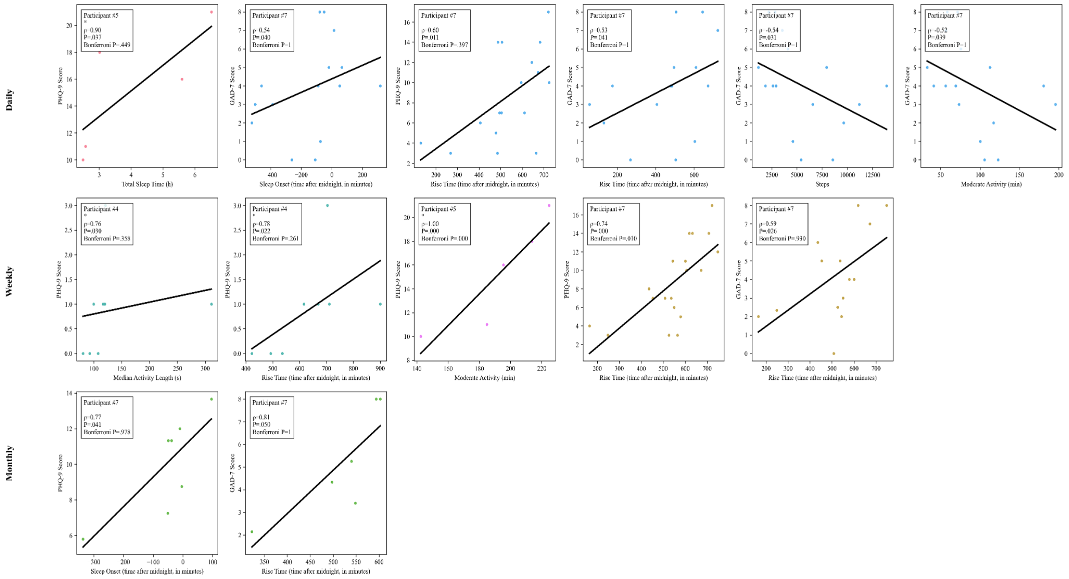

Figure S1. Complete Set of Significant Intra-Individual Associations Between Actigraphy Features and Questionnaire Scores Across Time Scales. * indicates fewer than 10 data points; Spearman correlation may be less reliable.

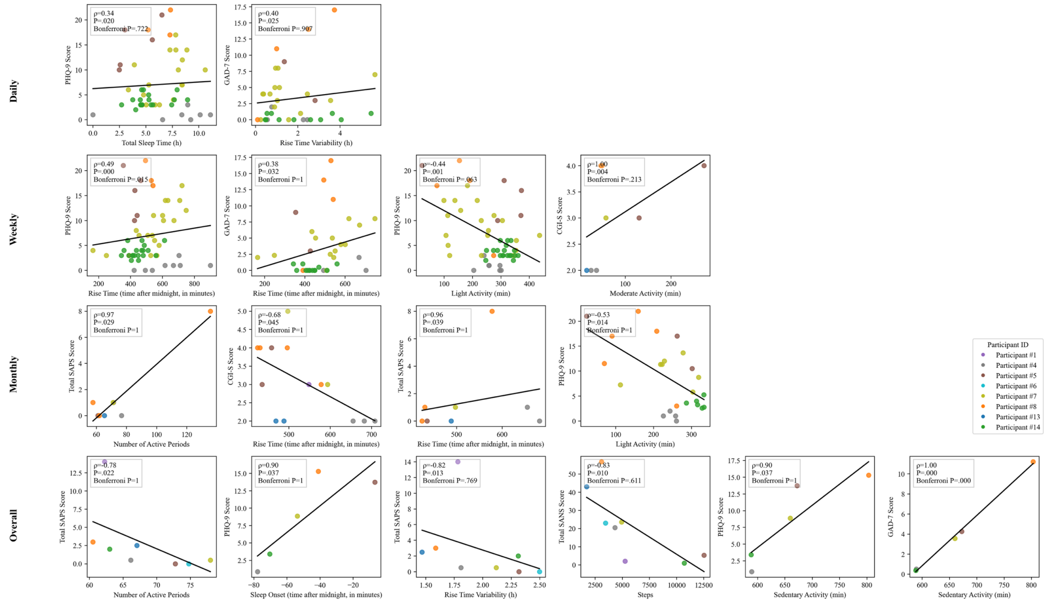

Figure S2. Complete Set of Significant Inter-Individual Associations Between Actigraphy Features and Questionnaire Scores Across Time Scales. Spearman correlations were used for overall averages across the full study duration. * indicates fewer than 10 data points; Spearman correlation may be less reliable.

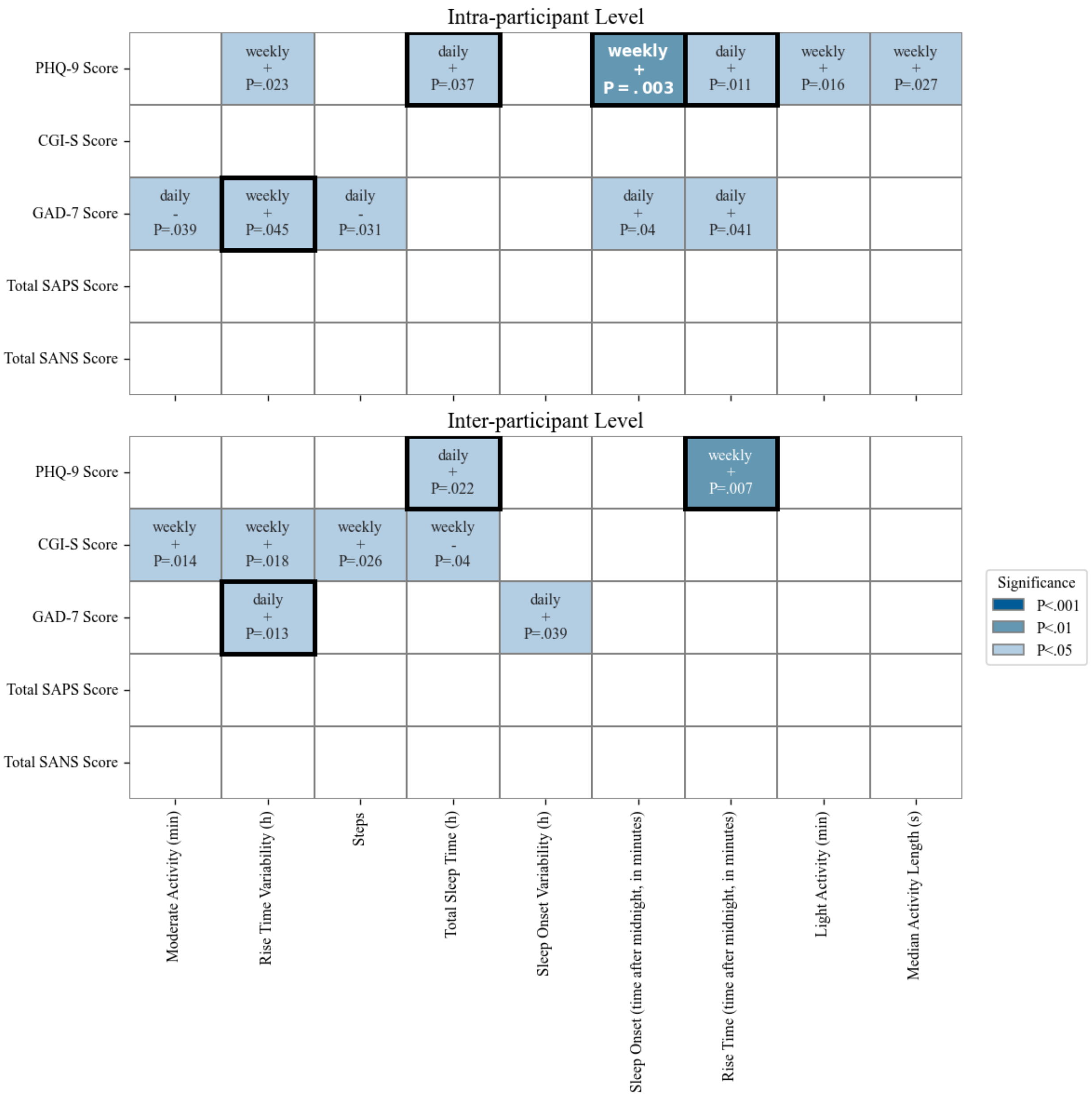

Figure S3. Heatmap of significant associations between actigraphy features and clinical questionnaires across multiple time scales at both intra- and inter-participant levels. "+" and "–" indicate the direction of the correlation. Cells with bold borders represent features with significant associations at multiple time scales (and/or across different participants for intra-individual analyses). Each cell displays only the most significant time scale. **This figure was generated using thresholds defining a valid week as >3 valid actigraphy days and a valid month as >10 valid days.**

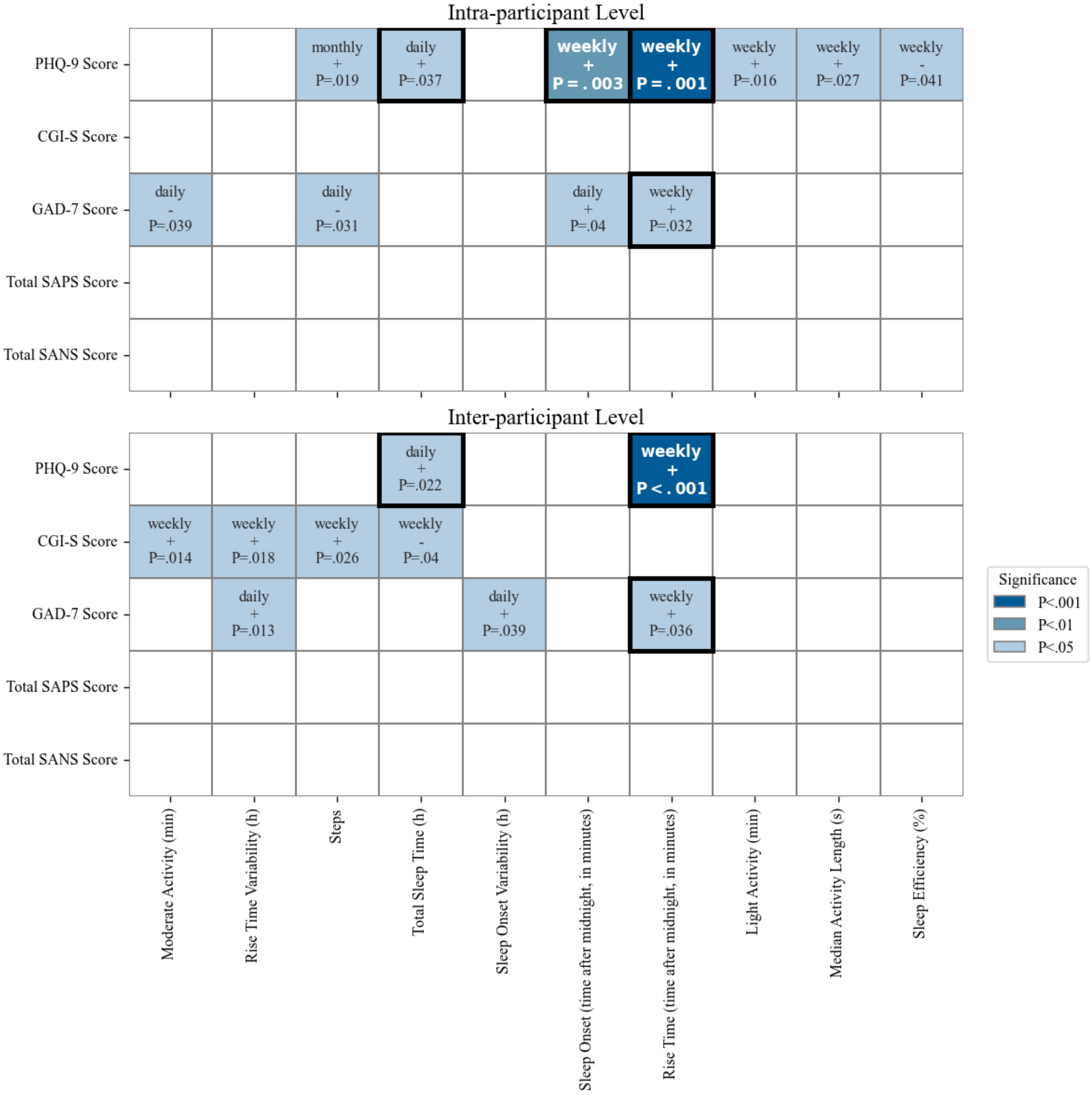

Figure S4. Heatmap of significant associations between actigraphy features and clinical questionnaires across multiple time scales at both intra- and inter-participant levels. "+" and "–" indicate the direction of the correlation. Cells with bold borders represent features with significant associations at multiple time scales (and/or across different participants for intra-individual analyses). Each cell displays only the most significant time scale. **This figure was generated using thresholds defining a valid week as >4 valid actigraphy days and a valid month as >15 valid days.**
